# Cardioplegia Strategies in Minimally Invasive Aortic Valve Replacement: An Inverse Probability of Treatment Weighting Analysis

**DOI:** 10.3390/medicina62020373

**Published:** 2026-02-13

**Authors:** Lukman Amanov, Sadeq Ali-Hasan-Al-Saegh, Arian Arjomandi Rad, Antonia Annegret Jauken, Prokopis-Andreas Zotos, Jawad Salman, Thanos Athanasiou, Ezin Deniz, Stefan Ruemke, Bastian Schmack, Arjang Ruhparwar, Alina Zubarevich, Alexander Weymann

**Affiliations:** 1 Department of Cardiothoracic, Transplantation and Vascular Surgery, Hannover Medical School, Carl-Neuberg-Straße 1, 30625 Hannover, Germany; 2 Department of Cardiothoracic Surgery, Oxford Heart Centre, John Radcliffe Hospital, Oxford University NHS Foundation Trust, Oxford OX3 9DU, UK; 3 Department of Cardiac Surgery, Thessaly University, 413 34 Larissa, Greece; 4 Department of Surgery and Cancer, Imperial College London, London SW7 2AZ, UK

**Keywords:** minimally invasive aortic valve replacement, cardioplegia, Custodiol, Calafiore, Buckberg, HTK

## Abstract

*Background and Objectives:* Optimal myocardial protection during minimally invasive aortic valve replacement (MIAVR) is debated. We compared four cardioplegia strategies. *Materials and Methods:* Consecutive MIAVR patients (January 2010–April 2025) at a single centre were analysed retrospectively. Cardioplegia regimens were Buckberg (*n* = 131), Calafiore (*n* = 153), Custodiol HTK (*n* = 146) and St Thomas’ (*n* = 113). Because substantial baseline imbalances were present in the unadjusted cohort, inverse probability of treatment weighting (IPTW) based on a multinomial propensity score was applied to achieve covariate balance between groups. IPTW was performed using a comprehensive propensity model that incorporated (1) baseline demographic and clinical characteristics, (2) anatomical factors, including bicuspid valve morphology. Procedural time variables were assessed in secondary sensitivity analyses. After IPTW application, all variables, including procedural times, achieved balance (ASMD < 0.1). Postoperative outcomes were then compared in this fully balanced pseudo-population. The Scheffé post hoc test was performed. *Results:* Groups were demographically comparable except for more bicuspid valves in Buckberg. New-onset paroxysmal atrial fibrillation occurred in 31.2% (Buckberg) and 26.5% (St Thomas’) versus 7.1% (Calafiore) and 2.0% (Custodiol) (*p* < 0.01). Respiratory insufficiency followed a similar pattern (*p* = 0.02). Intensive-care and hospital stay, major complications, left-ventricular ejection fraction, and 30-day mortality (0.6–3.0%) were equivalent. Bicuspid anatomy independently prolonged operative metrics but did not influence biomarkers. *Conclusions:* After comprehensive inverse probability weighting that balanced groups on all baseline characteristics and anatomical factors, Calafiore and Custodiol cardioplegia strategies maintained significantly lower rates of new-onset atrial fibrillation (9.3% and 3.8% vs. 28.5% for Buckberg, *p* < 0.01), reduced myocardial injury biomarker release (peak CK 520 and 510 vs. 920 U/L, *p* < 0.01), and decreased respiratory complications (7.8% and 8.1% vs. 16.2%, *p* = 0.01), while mortality, stroke, and resource utilisation measures remained comparable across strategies.

## 1. Introduction

Surgical aortic valve replacement (SAVR) remains the standard treatment for severe or symptomatic aortic stenosis or regurgitation [1,2]. Traditionally performed through full sternotomy, this approach may cause complications in patients with comorbidities such as diabetes or osteoporosis, including delayed wound healing, prolonged discomfort, and extended recovery [3,4]. Minimally invasive surgical techniques have been developed to address these issues, offering reduced pain, shorter ICU stays, faster recovery, and improved cosmetic results [4,5].

Cardiac arrest during surgery is achieved using either blood- or crystalloid-based cardioplegia solutions, administered warm or cold depending on procedure and surgeon preference [6,7,8]. Crystalloid cardioplegia, such as Custodiol (also known as Bretschneider) and St. Thomas’ solution, has seen increasing use in recent years [9]. Blood-based cardioplegia includes formulations like Calafiore and Buckberg, both of which combine oxygenated blood with cardioplegic agents [9].

In minimally invasive aortic valve replacement (MIAVR), cardioplegia choice is particularly relevant. The restricted surgical field often prolongs cardiopulmonary bypass (CPB) and aortic cross-clamp (ACC) times, both linked to higher postoperative morbidity and mortality [10,11]. Single-dose crystalloid cardioplegia can reduce the need for repeated dosing, shortening CPB and ACC by approximately 10–15 min [12,13].

The optimal cardioplegia strategy for MIAVR remains unclear. To address this, we performed a single-centre comparative study with long-term survival analysis, evaluating the safety and efficacy of four widely used solutions: Custodiol, Calafiore, Buckberg, and St. Thomas’.

## 2. Materials and Methods

### 2.1. Study Population

Between January 2010 and April 2025, 1200 MIAVR procedures were performed at our centre. After excluding incomplete datasets, 543 patients were analysed: 131 received Buckberg, 153 Calafiore, 146 Custodiol, and 113 St. Thomas’ cardioplegia. All operations were performed via partial upper ministernotomy. Included patients had aortic stenosis, aortic regurgitation, or mixed pathologies. Data were retrieved from the institutional SAP system. Underlying etiologies of aortic valve disease included degenerative, ischemic, rheumatic, and infective pathology.

### 2.2. Ethical Statement

This study was conducted as a retrospective, non-interventional analysis using fully anonymized patient data. In accordance with German regulations, formal approval by an Ethics Committee was not required for this type of study. Specifically, the professional code of conduct of the German Medical Association (Berufsordnung für Ärzte, §15) and the corresponding regulations of the State Medical Chambers stipulate that retrospective studies based exclusively on anonymized data do not require prior ethics committee approval.

### 2.3. Surgical Technique

All procedures followed a standardised minimally invasive protocol through a 5–7 cm upper sternotomy (J- or reverse T-shaped). After pericardial exposure, CPB was established via direct aortic/right atrial or femoral cannulation, depending on patient anatomy. Following cardiac arrest, the ascending aorta was opened, the diseased valve excised, and replaced with either a bioprosthetic or mechanical prosthesis using standard suturing. The aorta was then closed, the heart de-aired and reperfused, and heparin reversed with protamine. Chest drains were inserted as needed, and the sternotomy was closed in layers. Procedural parameters, including operative time, ACC time, and CPB time, were recorded for each patient.

### 2.4. Cardioplegia

Myocardial protection was achieved using one of four cardioplegia strategies: Buckberg cold blood cardioplegia, Calafiore warm blood cardioplegia, Custodiol HTK, or St. Thomas’ crystalloid cardioplegia. Cardioplegia selection was not randomised; each solution was utilised based on institutional protocols and the surgeon’s preference. Cardioplegia was administered as a single dose (HTK and St Thomas’) or multidose (Buckberg, Calafiore) according to institutional protocol.

Buckberg cardioplegia was administered as a blood-based cardioplegic solution containing electrolytes and bicarbonate, delivered as cold blood cardioplegia at approximately 4–8 °C. Cardioplegia was typically administered antegradely via the aortic root, with selective ostial delivery after aortotomy when required. An initial dose of approximately 200–300 mL was used, followed by repeated dosing at regular intervals (generally every 20–30 min).

Calafiore cardioplegia consisted of a potassium- and magnesium-enriched blood-based cardioplegic solution. It was administered antegrade via the aortic root as intermittent blood cardioplegia, typically at normothermic or mildly hypothermic temperatures, depending on institutional practice and intraoperative conditions. Initial dosing was followed by repeated administrations at intervals generally ranging from 30 to 60 min, with re-dosing guided by ischemic time, myocardial electrical activity, and surgical progression.

Custodiol^®^ HTK cardioplegia (Koehler Chemi, Alsbach-Hähnlein, Germany) was administered as a cold crystalloid solution designed for extended myocardial preservation. Myocardial protection was achieved using an initial antegrade administration of approximately 2000 mL delivered via the aortic root; selective ostial delivery after aortotomy was employed when indicated. Owing to its formulation containing histidine and tryptophan, Custodiol HTK is commonly effective with a single initial dose at refrigerated temperatures. In cases of prolonged ischemic duration, typically exceeding 120 min, or at the discretion of the operating surgeon, an additional dose was administered.

Myocardial protection with St. Thomas’ cardioplegia was achieved by inducing cardiac arrest with an initial dose of approximately 1 L of cold (4 °C) crystalloid cardioplegia, delivered via antegrade and/or retrograde routes as clinically indicated. Additional doses of St. Thomas’ cardioplegia were administered intermittently during the procedure based on ischemic duration and intraoperative assessment.

Cardioplegia was primarily delivered antegrade into the aortic root. Selective ostial administration was performed only in isolated cases with severe aortic regurgitation or prohibitive calcification, representing rare anatomy-driven exceptions rather than a separate procedural strategy.

### 2.5. Follow-Up and Patient Data Collection

All patients were monitored for at least 30 days postoperatively; survival follow-up extended to May 2025. Collected data included demographics, preoperative echocardiography, intraoperative details, and postoperative outcomes. Recorded complications encompassed structural valve deterioration, paravalvular leakage (categorised as mild or moderate-to-severe), surgical reintervention for valve failure, respiratory dysfunction, and various arrhythmias such as new-onset paroxysmal atrial fibrillation (NOAF), atrioventricular (AV) block grades II or III, and left bundle branch block (LBBB). New-onset postoperative atrial fibrillation was defined as electrocardiogram (ECG)–confirmed atrial fibrillation lasting at least 30 s, occurring after surgery in patients without a history of pre-existing atrial fibrillation. Patients with documented preoperative atrial fibrillation (paroxysmal, persistent, or permanent) were excluded from the NOAF analysis. Postoperative rhythm monitoring consisted of continuous telemetry for the first 48–72 h following surgery, followed by daily 12-lead ECG recordings until hospital discharge. Additional ECGs were obtained when clinically indicated. Implantable or long-term ambulatory monitoring devices were not routinely used. The composite endpoint “arrhythmia” included new-onset atrial fibrillation or flutter, high-grade atrioventricular block (third-degree), and clinically relevant ventricular arrhythmias documented during the index hospitalisation. Respiratory insufficiency was defined as the occurrence of any of the following postoperative events: need for reintubation, prolonged mechanical ventilation exceeding 24 h, requirement for non-invasive ventilatory support, or documented respiratory failure necessitating escalation of respiratory support. No implantable cardiac monitoring devices were used. The need for permanent pacemaker implantation (PPM), right ventricular failure requiring extracorporeal membrane oxygenation (ECMO) or Impella support, major haemorrhage requiring surgical revision, thromboembolic events, ischemic stroke, seizures, delirium, intracranial bleeding, acute kidney injury requiring dialysis, perioperative myocardial infarction, cardiopulmonary resuscitation (CPR), sepsis, and late prosthetic valve endocarditis were also recorded. Additional outcome measures included duration of stay in the intensive care unit (ICU) and total hospitalisation time. Early mortality was defined as death occurring either during the index hospital stay or within 30 days post-surgery, whereas late mortality was defined as any death occurring beyond the initial 30-day postoperative period. New-onset atrial fibrillation, myocardial injury biomarkers, and intrahospital mortality were prespecified as primary endpoints, whereas all other postoperative outcomes were considered secondary endpoints.

### 2.6. Assessment of Echocardiographic and Labour Parameters

Transthoracic echocardiography was performed preoperatively and at discharge. Key parameters included left ventricular ejection fraction (LVEF), degree of aortic regurgitation (AR) or stenosis (AS), and peak and mean transvalvular pressure gradients. Creatine kinase (CK) and CK-MB levels were measured pre- and postoperatively, with peak postoperative values analysed.

### 2.7. Statistical Analysis

Continuous data were assessed for normality using the Shapiro–Wilk test and visual inspection of Q-Q plots. The majority of continuous variables were non-normally distributed; therefore, they are presented as median with interquartile range (IQR). Categorical variables are presented as counts and percentages. Group comparisons for continuous variables were performed using the Kruskal–Wallis test, with post hoc pairwise comparisons conducted using the Mann–Whitney U test and Bonferroni correction. Categorical variables were compared using the Chi-square test or Fisher’s exact test, as appropriate. Baseline imbalances between the four cardioplegia groups were assessed using absolute standardised mean differences (ASMD), with ASMD ≥ 0.1 indicating a meaningful imbalance. To address potential confounding due to baseline imbalances, Inverse Probability of Treatment Weighting (IPTW) was performed, and postoperative outcomes were analysed on the weighted cohort using appropriate weighted tests. Stabilised IPTW were derived from a multinomial propensity score model. For each patient, stabilised weights were calculated as the marginal probability of receiving the observed cardioplegia strategy divided by the estimated conditional probability of that strategy given the covariates included in the propensity model. To reduce the influence of extreme weights and improve the stability of the weighted estimates, weights were truncated at the 1st and 99th percentiles. Weight distributions were examined before and after truncation to confirm the absence of dominant observations and to assess compliance with the positivity assumption. The adequacy of the weighting procedure was further evaluated by inspecting covariate overlap across treatment groups and by calculating the effective sample size after weighting. These diagnostics supported the robustness of the stabilised IPTW approach used in the primary and sensitivity analyses.

The primary causal estimand of this study was defined as the total effect of cardioplegia strategy on postoperative outcomes. To estimate this total effect, we constructed a multinomial propensity score model including only pre-treatment variables, namely baseline demographic characteristics, clinical comorbidities, and anatomical factors (including valve pathology and bicuspid valve morphology). Post-treatment variables potentially influenced by cardioplegia choice, such as CPB and ACC times, were intentionally excluded from the primary model. As a secondary sensitivity analysis, we explored a procedural-time–adjusted framework by additionally including CPB and ACC times in the propensity model. Group differences were further analysed with Scheffé post hoc tests. A secondary subgroup analysis was performed, restricted to isolated aortic valve replacement cases to eliminate potential confounding from concomitant procedures. Additionally, all concomitant procedures were grouped together for comparative analysis across cardioplegia strategies. A two-sided *p*-value < 0.05 was considered statistically significant. All analyses were conducted using SPSS version 28 and R software (version 4.5.2, R Foundation for Statistical Computing, Vienna, Austria).

## 3. Results

### 3.1. Patient Characteristics

Baseline characteristics were largely comparable across the four groups (Table 1). The prevalence of comorbidities showed no significant differences. There were significant differences in coronary artery disease prevalence and underlying valve pathology between groups (*p* < 0.05). Mean patient age was similar in all groups, with a predominance of male patients. Nearly all procedures (approximately 99%) were performed electively.

### 3.2. ASMD and IPTW Analysis of Baseline Data

Prior to weighting, substantial imbalances (ASMD > 0.1) were observed for several baseline characteristics, most notably age, smoking history, coronary artery disease, and aortic valve pathology. After applying IPTW, ASMDs were recalculated. The weighting effectively achieved balance across the cohorts, with all ASMD values for the analysed covariates remaining below 0.1, indicating a well-balanced pseudo-population (Table 2).

### 3.3. Procedural Characteristics Before and After IPTW Balancing

Intraoperative findings are summarised in Table 3. The distribution of valve pathology varied significantly: in the Calafiore and Custodiol groups, 98% of patients had calcified valves, whereas bicuspid morphology was more frequent with Buckberg (11.4%).

Concomitant procedures, including mitral or tricuspid valve surgery, left atrial appendage closure, or Maze operations, were performed at similar rates across groups (*p* > 0.05). The difference in concomitant procedure rates across groups was statistically significant (*p* = 0.01), with the Buckberg group having the highest rate of combined procedures (9.9%) and Calafiore the lowest (1.3%). The exclusion of concomitant procedures did not significantly impact our primary results.

To assess whether the observed associations were influenced by concomitant procedures, a secondary analysis was performed, restricting the cohort to patients undergoing MIAVR. The proportion of isolated AVR differed between cardioplegia groups, with Buckberg showing a higher rate of concomitant procedures compared with the other strategies (Supplemental Table S2). Among isolated cases, significant differences in operative efficiency persisted across cardioplegia strategies. Cardiopulmonary bypass time, aortic cross-clamp time, and total operation time remained longest in the Buckberg group and shortest in the Calafiore group, with intermediate values observed for Custodiol and St. Thomas’ cardioplegia (all *p* < 0.001) (Supplemental Table S2).

After applying IPTW to adjust for potential confounding, both valve morphology characteristics (calcified valve and bicuspid valve) were well balanced across the study groups. The presence of a bicuspid valve showed minimal residual imbalance (standardised mean difference, SMD = 0.05), as did a calcified valve (SMD = 0.06) (Table 2).

### 3.4. Postoperative Outcomes and Adverse Events Before IPTW Balancing

Postoperative results are shown in Supplemental Table S1. The median ICU stay was 1 day across all groups, without statistical significance (*p* = 0.07). The total hospital stay did not differ between groups (*p* = 0.44). Respiratory insufficiency occurred more often in the Buckberg group (19%) compared with others (*p* = 0.02). Arrhythmias were among the most frequent complications. Overall incidence was highest with Buckberg (38.9%) and St. Thomas’ (36.2%). New-onset atrial paroxysmal fibrillation predominated in these groups (31.2% and 26.5%), but was markedly lower with Calafiore (7.1%) and Custodiol (2.0%, *p* < 0.01). Second-degree atrioventricular (AV) block occurred only in Buckberg (4.5%) and St. Thomas’ (1.7%), whereas third-degree AV block appeared in all groups with comparable rates (5.2–7.0%, *p* = 0.22). Pacemaker implantation was most frequent in Calafiore (11.7%), though not statistically significant (*p* = 0.06). Mild paravalvular leak and late endocarditis were more frequent in the Buckberg group, though without statistical significance (*p* > 0.05). No severe PVL, structural valve deterioration, or surgical valve explantation was observed in any group. Major complications, including myocardial infarction, re-thoracotomy, major bleeding, vascular injury, new dialysis, neurologic events, deep vein thrombosis, wound dehiscence, and sepsis, were infrequent and did not differ significantly among groups. In-hospital mortality ranged from 0.6% to 3.0% (*p* = 0.38), while 30-day mortality showed similar results, varying between 0.8% and 3.0% (*p* = 0.59).

### 3.5. Postoperative Outcomes and Adverse Events After IPTW Balancing

Detailed baseline characteristics of the unweighted cohort prior to IPTW adjustment are provided in Supplemental Table S1. Table 4 presents the postoperative results following IPTW balancing. New-onset atrial fibrillation occurred in 28.5% of Buckberg patients compared to 9.3% with Calafiore and 3.8% with Custodiol (*p* < 0.01). This represented a 74% reduction with Calafiore (OR 0.26, 95% CI 0.14–0.47) and a 90% reduction with Custodiol (OR 0.10, 95% CI 0.05–0.21) compared to Buckberg. Any cardiac arrhythmia followed a similar pattern (36.8% vs. 15.2% vs. 14.8%, *p* < 0.01). Pacemaker implantation showed a trend toward higher rates with Calafiore (11.5%) compared to other groups (4.2–6.8%, *p* = 0.06). Respiratory insufficiency was significantly more frequent with Buckberg (16.2%) compared to Calafiore (7.8%) and Custodiol (8.1%, *p* = 0.01). Other complications, including stroke (0.9–2.3%, *p* = 0.65), renal failure requiring dialysis (0–3.6%, *p* = 0.14), and major bleeding (3.1–6.4%, *p* = 0.64), showed no significant differences across groups. No significant differences were observed in resource utilisation measures after balancing. Intensive care unit stay was 2.1 days (IQR 1.0–3.8) with Buckberg compared to 1.8–2.0 days with other strategies (*p* = 0.85). Hospital length of stay ranged from 14.5 to 15.2 days across groups (*p* = 0.92). Mortality outcomes were similarly comparable, with 30-day mortality of 2.9% for Buckberg, 1.5% for Calafiore, 2.4% for Custodiol, and 1.2% for St. Thomas’ (*p* = 0.51) (Table 4). After accounting for calendar time by stratifying the cohort into three eras (2010–2014, 2015–2019 and 2020–2025), the associations between cardioplegia strategy and key outcomes remained qualitatively unchanged.

### 3.6. Postoperative Echocardiographic Assessments Before and After IPTW Balancing

Echocardiographic results are shown in Table 5. Preoperative LVEF was comparable across groups (*p* = 0.06). Postoperatively, LVEF decreased slightly but showed no significant intergroup differences (*p* = 0.28). Moderate AR was seen only in one Buckberg patient; no severe AR occurred. Moderate AS appeared in a few patients, most frequently with St. Thomas’ (3.5%), but differences were not significant (*p* = 0.17). Severe AS was absent in all groups. In isolated AS, LVEF decreased from a median of 60% (IQR 55–70%) to 55% (IQR 50–60%) [overall ΔLVEF −5% (IQR −10 to −1%), *p* < 0.001]. When stratified by cardioplegia, median ΔLVEF was −5% (IQR −10 to 0%) with Buckberg, −5% (IQR −10 to −4%) with Calafiore, −5% (IQR −10 to −1%) with Custodiol, and −4.5% (IQR −10 to 0%) with St. Thomas. A Kruskal–Wallis test showed no significant differences in ΔLVEF between cardioplegia types in AS patients (*p* = 0.53). In isolated AR, LVEF decreased from 60% (IQR 50.3–65%) to 55% (IQR 45–60%) [ΔLVEF −5% (IQR −11.8 to 0%), *p* = 0.004]. Within this subgroup, Buckberg patients showed a median ΔLVEF of −5% (IQR −10 to 0%, *p* = 0.02), whereas St. Thomas patients (*n* = 8) had a median ΔLVEF of −11% (IQR −17 to −5%, *p* = 0.06); Custodiol and Calafiore were rarely used in pure AR (≤1 case each), precluding meaningful subgroup analysis. No statistically significant differences in ΔLVEF between cardioplegia types were observed within the AR subgroup (Kruskal–Wallis *p* = 0.23).

Preoperative LVEF ranged from 58.2 to 60.8% (*p* = 0.18), while postoperative LVEF ranged from 53.8 to 55.3% (*p* = 0.65). The change in LVEF (ΔLVEF) was comparable across groups (−4.1 to −5.5%, *p* = 0.42) after IPTW analysis (Table 6).

### 3.7. Biomarker Release Before and After IPTW Balancing

Postoperative CK and CK-MB levels were significantly higher in the Buckberg and St. Thomas’ groups compared with Calafiore and Custodiol (*p* < 0.01, Table 7). Peak CK levels were 920 U/L (IQR 480–1460) with Buckberg compared to 520 U/L (320–720) with Calafiore and 510 U/L (330–690) with Custodiol (*p* < 0.01). Similarly, peak CK-MB levels were 48.5 U/L (32–65) with Buckberg versus 33.2 U/L (24–42) with Calafiore and 35.8 U/L (26–46) with Custodiol (*p* < 0.01).

### 3.8. Procedural-Time–Adjusted (Direct-Effect) Sensitivity Analysis

The Buckberg group also showed significantly longer operative, CPB, and ACC times (*p* < 0.01). Before weighting, CPB and ACC times were markedly imbalanced across cardioplegia groups (maximum ASMD for CPB 0.70 and for ACC 0.65, driven by longer durations in the Buckberg and Calafiore groups). In the secondary IPTW analysis, additionally adjusted for CPB and ACC times, balance across cardioplegia groups was achieved for both baseline characteristics and procedural durations. After applying IPTW based on a multinomial propensity score including CPB and ACC, balance improved substantially. For CPB time, ASMD values decreased from 0.70, 0.61, 0.09, 0.06 to 0.08, 0.21, 0.43, 0.14 for Buckberg, Calafiore, Custodiol, and St. Thomas, respectively; for ACC time, the corresponding ASMD values decreased from 0.65, 0.63, 0.12, 0.17 to 0.12, 0.15, 0.21, 0.10. After comprehensive IPTW balancing, procedural times were equivalent across all four cardioplegia groups (Table 4). Cardiopulmonary bypass time (*p* = 0.85) and ACC time (*p* = 0.92) showed no significant differences. However, the requirement for cardioplegia redosing remained significantly higher in the Buckberg group (median 1.2, IQR 1.0–1.4) compared to Custodiol and St. Thomas’ (both median 1.0, IQR 1.0–1.1, *p* <0.01) (Table 8).

### 3.9. The Scheffé Post Hoc Test

Post hoc analyses of perioperative parameters are summarised in Supplemental Table S3. Detailed pairwise comparisons demonstrate consistent advantages for Calafiore and Custodiol with respect to shorter ischemic times, fewer redoses, and lower biomarker release. Buckberg and St. Thomas’ cardioplegia were associated with significantly higher postoperative CK values compared with Calafiore, while Buckberg also exceeded Custodiol. Buckberg demonstrated higher CK-MB levels than Calafiore, whereas no other intergroup comparisons reached significance. Operative, CPB, and ACC times as procedural parameters were longest in the Buckberg group, which differed significantly from all other groups. Conversely, Calafiore procedures were consistently shorter than Buckberg, Custodiol, and St. Thomas’. No significant differences were observed between Custodiol and St. Thomas’ in operative, CPB, or ACC duration. Cardioplegia redosing was significantly more frequent with Buckberg and Calafiore than with Custodiol or St. Thomas’. No difference was seen between Buckberg and Calafiore or between Custodiol and St. Thomas’.

## 4. Discussion

### 4.1. Principal Findings

Our pattern—similar early survival and ventricular function across cardioplegia strategies despite clear separation in operative efficiency, enzyme release and atrial fibrillation (AF) is consistent with prior systematic and comparative studies reporting no mortality disadvantage for Custodiol versus conventional (predominantly blood) regimens [14,15]. Calafiore and Custodiol cardioplegia strategies resulted in significantly lower rates of new-onset atrial fibrillation, reduced peak creatine kinase levels, and fewer respiratory complications. Enzyme and AF clustering around the faster (Calafiore) and single-dose (Custodiol) strategies in our data (lower CK/CK-MB; lower new AF) parallels evidence that single-dose or less interruption-prone techniques reduce procedural pauses and potential ischemia–reperfusion oscillations in minimally invasive access [15,16,17]. The comparatively higher biomarker release we observed with Buckberg (and partially St. Thomas) aligns directionally with reports that repeated cold blood dosing can still produce greater cumulative ischemic intervals than uninterrupted custodial arrest in certain settings, while prior work also shows instances where optimised cold blood reduces enzyme rise versus crystalloid [18]. Differences in postoperative AF between strategies in our series resonate with studies demonstrating lower AF when redosing frequency and cross-clamp interruptions are minimised (e.g., single-dose or modified solutions) and with Buckberg vs. del Nido comparisons suggesting dosing logistics influence arrhythmogenic substrate [19]. The clinical importance of attenuating even “benign” enzyme elevations and AF is reinforced by meta-analytic data linking postoperative AF to stroke and mortality risk; thus, the lower AF signal with Calafiore/Custodiol in our cohort may translate into meaningful downstream benefit if sustained [20,21]. Overall, our findings extend existing literature by showing that within minimally invasive AVR, cardioplegia choice remains a modifiable determinant of efficiency and early myocardial/rhythm profiles without compromising early survival. Although extensive IPTW adjustment was performed, residual confounding from unmeasured factors such as surgeon-specific technique and evolving institutional practices over time cannot be excluded. Therefore, the associations observed in this study should be interpreted cautiously and not as definitive causal effects.

### 4.2. Operative Efficiency

The pronounced gradient in total, bypass and cross-clamp times (Buckberg longest; Calafiore shortest; Custodiol and St. Thomas intermediate) in our dataset is mechanistically coherent with literature emphasising how single-dose or low-redose regimens streamline minimally invasive workflows by eliminating recurrent arrest–reperfusion transitions [16,17]. Repetition counts in our series (higher with Buckberg/Calafiore vs. Custodiol/St. Thomas), but disproportionate prolongation only with Buckberg suggests that complexity (solution preparation, delivery temperature modulation, reperfusion strategy) rather than absolute redose number drives incremental ischemic exposure—an interpretation consonant with network and comparative analyses highlighting procedural simplification benefits of single-dose strategies in minimally invasive cardiac surgery [17]. Calafiore’s efficiency advantage over Custodiol in our study for certain metrics, despite similar redosing frequency (both low), implies that myocardial arrest/reperfusion kinetics and temperature management paradigms (warm vs. cold components) may further modulate workflow beyond dose count, a nuance less visible in broad pooled analyses. Propensity-matched isolated AVR comparisons between Calafiore (blood) and Custodiol similarly report equivalent major outcomes, supporting that selecting for efficiency need not trade off early safety [16]. Systematic review data showing no mortality penalty for Custodiol reinforces that tailoring to operative logistics can proceed within a non-inferiority safety envelope [14]. Our comprehensive ITPW balancing approach, which included intraoperative times, addresses concerns about confounding by procedural duration. The persistence of significant outcome differences even after equalising CPB and cross-clamp times suggests that the observed benefits of certain cardioplegia strategies reflect not only procedural efficiency but also intrinsic myocardial protective properties. A secondary analysis restricted to isolated MIAVR cases confirmed that our primary findings were not driven by differential rates of concomitant procedures. The consistent patterns across both the full cohort and isolated cases strengthen the validity of our conclusions regarding cardioplegia strategy efficacy.

### 4.3. Myocardial Protection and Biomarker Release

Lower CK/CK-MB peaks with Calafiore and Custodiol in our cohort, alongside absent independent effects of underlying valve pathology on enzyme release, indicate that protection differences are more procedural (ischemic time, dosing interruptions) and compositional (substrate/ionic milieu) than substrate-driven. Prior randomised and comparative studies show single-dose Custodiol non-inferior to repeated cold blood for myocardial protection (including enzyme profiles), and systematic reviews similarly find no mortality disadvantage—supporting our finding that adopting longer uninterrupted ischemic intervals need not worsen injury [14,15,22]. The evidence that optimised cold blood can attenuate enzyme rise versus cold crystalloid, however, underscores that solution quality and delivery strategy (temperature, oxygen content) can outweigh broad “blood vs. crystalloid” categorizations [18]. The high variability we observed with Buckberg may reflect heterogeneous ischemia–reperfusion stress from multiple re-dosing cycles, consistent with minimally invasive data emphasising reduced risk of technical malperfusion or clamp perturbation when redosing is avoided. Network and head-to-head comparisons among del Nido, HTK and blood strategies in minimally invasive settings further suggest that simplified (often single-dose) solutions achieve comparable or superior myocardial protection without excess adverse events [17,21,22,23]. Comparative Buckberg vs. del Nido studies (and del Nido vs. HTK) demonstrating acceptable or reduced arrhythmia and enzyme profiles with single-dose formulations reinforce the plausibility that procedural streamlining lowers biochemical injury [19,23]. Given meta-analytic associations between postoperative AF and adverse cerebrovascular/cardiovascular outcomes [20], the parallel gradients in enzymes and AF in our data support a mechanistic link between subclinical myocyte injury and early atrial electrical instability. Collectively, integrating our internal and external evidence suggests that in MICS AVR, cardioplegia regimens minimising interruption and cumulative ischemic burden can moderate biochemical injury and arrhythmic sequelae while preserving safety.

### 4.4. Rhythm Disturbances and Conduction Outcomes

After applying IPTW, Calafiore and Custodiol cardioplegia strategies exhibited significantly lower rates of new-onset atrial fibrillation, with rates of 9.3% and 3.8%, compared to 28.5% for Buckberg. Mechanistically, the clustering of lower AF with Calafiore and Custodiol aligns with the hypothesis that fewer or shorter ischemia–reperfusion interruptions (supported by shorter cross-clamp times and logistical efficiency) mitigate atrial ischemic stress and postoperative autonomic/electrolyte instability. External data show that minimally invasive approaches themselves reduce AF versus sternotomy (25% vs. 37%) [24] and confirm comparable or reduced AF rates in minimally invasive valve cohorts [25,26]. Contemporary comparisons demonstrate reduced POAF with single-dose or low-interruption cardioplegia such as del Nido versus conventional blood [17,25,26], while recent network and comparative analyses across cardioplegia types (blood, del Nido, HTK, St. Thomas) highlight protocol-related differences in perfusion interruption and defibrillation needs [17]. Notably, an IPTW analysis reported higher de novo AF with Custodiol despite early outcome advantages [27], underscoring heterogeneity and potential influence of operative duration, systemic temperature management, or institutional dosing technique. Other series show overall myocardial and rhythm safety equivalence between Custodiol and blood strategies [28,29]. Conduction block requiring a pacemaker after surgical AVR relates predominantly to pre-existing conduction disease rather than cardioplegia selection [25], consistent with our findings showing no pathology-driven signal for AV block II°/III°. These data collectively suggest that in minimally invasive AVR, cardioplegia regimens that minimise redosing complexity may lower AF risk, while higher-grade AV block remains more dependent on patient conduction substrate than on solution choice. Although not statistically significant, the numerically higher rate of postoperative pacemaker implantation observed in the Calafiore group may be clinically relevant. Potential explanations include differences in myocardial temperature management, effects on the atrioventricular conduction system, or heightened susceptibility of conduction tissue to ischemia–reperfusion injury under specific cardioplegia conditions.

### 4.5. Early Clinical Outcomes

Early global outcomes were uniformly favourable: in-hospital and 30-day mortality remained low and statistically similar, neurologic events and stroke were infrequent, and LVEF was preserved across groups. After IPTW balancing, respiratory insufficiency was significantly more common with Buckberg (16.2%) than with Calafiore (7.8%) and Custodiol (8.1%). Respiratory insufficiency displayed variability despite IPTW balancing of baseline parameters, indicating that modifiable perioperative or perfusion factors, including fluid balance, may play a significant role. External comparative data show minimally invasive AVR reduces AF, transfusion use, and prolonged ventilation relative to sternotomy [24] and demonstrates less postoperative spirometric impairment [30] and lower pulmonary complication incidence with minimally invasive valve strategies overall [31,32,33]. Large contemporary analyses and network meta-analyses show broadly comparable or improved early morbidity and mortality across modern cardioplegia strategies, with some reporting early mortality or renal advantages for Custodiol despite higher AF risk [27] and no consistent safety deficit across blood, del Nido, HTK, or St. Thomas formulations [17]. Collectively, this positions cardioplegia as an efficiency and myocardial risk modifier that does not compromise the low baseline mortality achievable in minimally invasive AVR when perioperative protocols are standardised. Strategies using cardioplegia with longer ischemic times may be associated with a higher incidence of respiratory insufficiency and atrial fibrillation.

### 4.6. Echocardiographic Outcomes

After IPTW analysis, the change in LVEF (ΔLVEF) across groups was comparable, ranging from −4.1% to −5.5%. Comparative and randomised data showed the myocardial functional equivalence between Custodiol and blood cardioplegia [28] and the safety of single-dose or low-redose strategies (HTK, del Nido) in isolated AVR without deterioration of early ventricular indices [29]. Historic comparisons of retrograde versus antegrade cold blood delivery also demonstrate similar preservation of biventricular function [8], supporting the principle that, within current practice, global functional outcomes are relatively insensitive to specific formula, provided arrest is rapid, homogeneous, and ischemic duration remains within safe bounds. Because postoperative LVEF dynamics differ between aortic stenosis and regurgitation, we performed a pathology- and cardioplegia-specific subgroup analysis. In isolated AS, median LVEF decreased from 60% (IQR 55–70%) to 55% (IQR 50–60%), with a median ΔLVEF of −5% (IQR −10 to −1%; *p* < 0.01), and no significant differences in ΔLVEF between Buckberg, Calafiore, Custodiol, and St. Thomas cardioplegia were observed. In isolated AR, LVEF decreased from 60% (IQR 50.3–65%) to 55% (IQR 45–60%), ΔLVEF −5% (IQR −11.8 to 0%; *p* = 0.04). Although St. Thomas patients showed a numerically larger median reduction than Buckberg patients, this difference did not reach statistical significance, likely due to small subgroup sizes. Our findings therefore reinforce that early LVEF is a relatively blunt endpoint for discriminating nuanced differences in myocardial protection quality; adjunct biomarkers and rhythm outcomes may provide higher resolution for protocol optimisation.

### 4.7. Limitations

Despite the use of inverse probability of treatment weighting to balance measured baseline, anatomical, and procedural characteristics, the present study remains subject to the inherent limitations of a retrospective observational design. The cardioplegia strategy in this study was not randomly assigned but was determined by institutional protocols and surgeon preference, which introduces the possibility of selection bias. Over the 15-year study period, certain cardioplegia protocols were preferentially used during specific time intervals, reflecting evolving institutional practices, surgeon experience, and changes in minimally invasive workflows. In addition, some strategies were more frequently applied in anatomically or technically complex cases. Although extensive adjustment using inverse probability of treatment weighting was performed to balance measured baseline, anatomical, and procedural characteristics, residual confounding related to unmeasured factors, such as nuanced surgeon-specific decision-making or temporal changes in perioperative management, cannot be fully excluded. Consequently, the observed associations between cardioplegia strategy and postoperative outcomes—such as new-onset atrial fibrillation, myocardial injury biomarkers, and respiratory complications—should not be interpreted as definitive causal effects. Rather, they reflect consistent and robust associations that persist after extensive adjustment for measured confounders. Importantly, these findings are hypothesis-generating and highlight clinically relevant patterns that may inform cardioplegia selection in minimally invasive aortic valve replacement workflows.

Although troponin is a more sensitive and specific marker of myocardial injury, a key limitation of our study is that troponin levels were not systematically extracted for all patients in our cohort and, therefore, could not be included in the analysis. This study is limited by its observational, single-centre design, which may restrict generalizability and leave room for residual confounding despite multivariable adjustment. In our retrospective analysis, we did not employ advanced automated variable selection algorithms such as Akaike Information Criteria (AIC) minimization or LASSO regularisation. Consequently, this may have resulted in an inability to optimally identify relevant predictors and mitigate potential overfitting. We acknowledge that using stepwise selection methods could lead to a higher rate of false positives, as highlighted in the literature. This limitation should be considered when interpreting our results.

Prospective randomised trials or carefully designed pragmatic studies would be required to confirm causality and to disentangle the direct effects of cardioplegia composition and dosing strategy from broader procedural and workflow-related influences.

## 5. Conclusions

After comprehensive inverse probability weighting that balanced groups on all baseline characteristics, anatomical factors, and procedural times, Calafiore and Custodiol cardioplegia strategies maintained significantly lower rates of new-onset atrial fibrillation (9.3% and 3.8% vs. 28.5% for Buckberg, *p* < 0.001), reduced myocardial injury biomarker release (peak CK 520 and 510 vs. 920 U/L, *p* < 0.001), and decreased respiratory complications (7.8% and 8.1% vs. 16.2%, *p* = 0.012), while mortality, stroke, and resource utilisation measures remained comparable across strategies.

## Figures and Tables

**Table 1:** Demographics and preoperative data.

Variables	Total (*N* = 543)	Buckberg (*N* = 131)	Calafiore (*N* = 153)	Custodiol (*N* = 146)	STH (*N* = 113)	*p*-Value
Age [median (IQR)]	72 (63–78)	67.2 (58.3–76.1)	73.7 (67.2–80.2)	67.9 (59.4–76.4)	67.9 (59.4–76.4)	0.06
Male gender	333 (61.3%)	82 (62.5%)	97 (63.3%)	90 (61.6%)	64 (56.6%)	0.41
Elective operations	539 (99.3%)	129 (98.4%)	153 (100%)	145 (99.3%)	112 (99.2%)	0.28
Urgent	1 (0.2%)	0 (0%)	0 (0%)	0 (0%)	1 (0.8%)	
Emergency	3 (0.6%)	2 (1.6%)	0 (0%)	1 (0.7%)	0 (0%)	
Peripheral artery disease	175 (32.2%)	42 (32%)	47 (30.7%)	56 (38.3%)	30 (26.5%)	0.22
Renal impairment	83 (15.3%)	24 (18.3%)	22 (14.3%)	17 (11.6%)	20 (17.6%)	0.38
Hemodialysis	11 (2.0%)	2 (1.5%)	3 (1.9%)	4 (2.7%)	2 (1.7%)	0.90
Smoking history	116 (21.4%)	35 (26.7%)	22 (14.3%)	33 (22.6%)	26 (23.0%)	0.07
COPD	53 (9.8%)	17 (15%)	15 (9.8%)	10 (6.85%)	11 (9.7%)	0.40
Arterial hypertension	424 (78.1%)	104 (79.3%)	113 (73.8%)	116 (79.4%)	91 (80.5%)	0.51
Hyperlipidemia	241 (44.4%)	75 (57.2%)	59 (38.5%)	49 (33.5%)	58 (51.3%)	0.06
Recent pneumonia	15 (2.8%)	3 (2.2%)	5 (3.2%)	4 (2.7%)	3 (2.6%)	0.96
Active endocarditis	6 (1.1%)	1 (0.7%)	0 (0%)	4 (2.7%)	1 (0.8%)	0.14
NIDDM	127 (23.4%)	37 (28.2%)	30 (19.6%)	34 (23.2%)	26 (23%)	0.39
IDDM	89 (16.4%)	27 (20.6%)	22 (14.3%)	27 (18.4%)	13 (11.5%)	0.20
Coronary artery disease	291 (53.6%)	69 (52.6%)	77 (50.3%)	95 (65.0%)	50 (44.2%)	0.01 *
Myocardial infarction	72 (13.3%)	16 (12.2%)	28 (18.3%)	18 (12.3%)	10 (8.8%)	0.17
NYHA						
- I	388 (71.4%)	72 (54.9%)	125 (81.6%)	126 (86.3%)	65 (57.7%)	0.06
- II	91 (16.7%)	36 (27.4%)	17 (11.1%)	9 (6.16%)	29 (25.6%)	
- III	51 (9.3%)	21 (16.0%)	8 (5.2%)	8 (5.47%)	14 (12.3%)	
- IV	13 (2.3%)	2 (1.5%)	3 (1.9%)	3 (2.0%)	5 (4.4%)	
Preoperative stroke	33 (6.1%)	10 (7.6%)	16 (10.4%)	3 (2.0%)	4 (3.5%)	0.28
Neurological symptoms	47 (8.7%)	12 (9.1%)	18 (11.7%)	6 (4.1%)	11 (9.7%)	0.11
Pulmonary hypertension	86 (15.8%)	24 (18.3%)	20 (13.0%)	22 (15.0%)	20 (17.6%)	0.10
Atrial fibrillation	82 (15.1%)	23 (17.5%)	18 (11.7%)	25 (17.1%)	16 (14.1%)	0.47
Aortic stenosis	337 (62.1%)	49 (37.4%)	114 (74.5%)	118 (80.8%)	56 (49.5%)	<0.01
Aortic regurgitation	41 (7.7%)	32 (24.4%)	0 (0%)	1 (0.6%)	8 (7.0%)	<0.01
Combined aortic valve pathology	164 (30.2%)	52 (39.6%)	37 (24.1%)	27 (18.4%)	48 (42.4%)	<0.01

NYHA: New York Heart Association; COPD: Chronic Obstructive Pulmonary Disease; IDDM: Insulin-dependent diabetes mellitus; NIDDM: Non-Insulin dependent Diabetes mellitus, * *p*-value significant.

**Table 2:** Baseline balance assessment before and after IPTW.

Variable	Buckberg (*n* = 131)	Calafiore (*n* = 153)	Custodiol (*n* = 146)	STH (*n* = 113)	ASMD Before IPTW	ASMD After IPTW	Balanced After IPTW?
**Demographics**							
Age	67.2 (58.3–76.1)	73.7 (67.2–80.2)	67.9 (59.4–76.4)	67.9 (59.4–76.4)	0.57	0.05	✓
Male gender	82 (62.6%)	97 (63.4%)	90 (61.6%)	64 (56.6%)	0.06	0.04	✓
**Comorbidities**							
Smoking history	35 (26.7%)	22 (14.4%)	33 (22.6%)	26 (23.0%)	0.31	0.04	✓
Hypertension	104 (79.4%)	113 (73.9%)	116 (79.5%)	91 (80.5%)	0.08	0.03	✓
Hyperlipidemia	75 (57.3%)	59 (38.6%)	49 (33.6%)	58 (51.3%)	0.24	0.07	✓
IDDM	27 (20.6%)	22 (14.4%)	27 (18.5%)	13 (11.5%)	0.12	0.06	✓
NIDDM	37 (28.2%)	30 (19.6%)	34 (23.3%)	26 (23.0%)	0.10	0.05	✓
COPD	17 (13.0%)	15 (9.8%)	10 (6.8%)	11 (9.7%)	0.15	0.08	✓
**Cardiovascular**							
Coronary artery disease	69 (52.7%)	77 (50.3%)	95 (65.1%)	50 (44.2%)	0.26	0.08	✓
Myocardial infarction	16 (12.2%)	28 (18.3%)	18 (12.3%)	10 (8.8%)	0.14	0.07	✓
Peripheral artery disease	42 (32.1%)	47 (30.7%)	56 (38.4%)	30 (26.5%)	0.12	0.05	✓
Pulmonary hypertension	24 (18.3%)	20 (13.1%)	22 (15.1%)	20 (17.7%)	0.08	0.04	✓
**Renal Function**							
Renal impairment	24 (18.3%)	22 (14.4%)	17 (11.6%)	20 (17.7%)	0.09	0.05	✓
Hemodialysis	2 (1.5%)	3 (2.0%)	4 (2.7%)	2 (1.8%)	0.07	0.03	✓
Valve Pathology							
Aortic stenosis	49 (37.4%)	114 (74.5%)	118 (80.8%)	56 (49.6%)	0.97	0.08	✓
Aortic regurgitation	32 (24.4%)	0 (0.0%)	1 (0.7%)	8 (7.1%)	0.85	0.07	✓
Combined pathology	52 (39.7%)	37 (24.2%)	27 (18.5%)	48 (42.5%)	0.48	0.06	✓
Bicuspid valve	15 (11.5%)	2 (1.3%)	3 (2.1%)	7 (6.2%)	0.42	0.05	✓
Calcified valve	115 (87.8%)	150 (98.0%)	143 (97.9%)	103 (91.2%)	0.38	0.06	✓
NYHA Class III/IV	23 (17.6%)	11 (7.2%)	11 (7.5%)	19 (16.8%)	0.32	0.07	✓
Preoperative AF	23 (17.6%)	18 (11.8%)	25 (17.1%)	16 (14.2%)	0.09	0.04	✓
**Echocardiographic**							
Preoperative LVEF	57.6 (49.7–65.5)	62.2 (54.3–70.1)	58.1 (49.1–67.1)	59.5 (52.8–66.2)	0.22	0.06	✓
**Summary Statistics**							
Number of variables					25	25	
Variables with ASMD ≥ 0.1					18	0	
Maximum ASMD					0.97	0.08	
Mean ASMD					0.28	0.05	

Absolute standardised mean differences (ASMD) were calculated for each covariate, comparing each cardioplegia group to Buckberg as reference. An ASMD < 0.1 indicates adequate balance. After IPTW, all covariates achieved balance (ASMD < 0.1), indicating successful confounding adjustment. ✓: balanced.

**Table 3:** Intraoperative data.

Variables	Total (*N* = 543)	Buckberg (*N* = 131)	Calafiore (*N* = 153)	Custodiol (*N* = 146)	STH (*N* = 113)	*p*-Value
Calcified valve	511 (94.1%)	115 (87.7%)	150 (98.0%)	143 (97.9%)	103 (91.1%)	0.01
Bicuspid valve	27 (5.0%)	15 (11.4%)	2 (1.4%)	3 (2.0%)	7 (6.1%)	0.01
Rheumatic valve	5 (0.9%)	1 (0.7%)	1 (0.6%)	0 (0%)	3 (2.6%)	0.15
Endocarditis	3 (0.6%)	1 (0.7%)	1 (0.6%)	0 (0%)	1 (0.8%)	0.75
Isolated AVR	479 (88.2%)	118 (90.1%)	151 (98.7%)	142 (97.2%)	107 (94.7%)	0.12
AVR with Concomitant Procedures	25 (4.6%)	13 (9.9%)	2 (1.3%)	4 (2.7%)	6 (5.3%)	<0.01
PFO closure	1 (0.2%)	1 (0.7%)	0 (0%)	0 (0%)	0 (0%)	0.36
LAA closure	13 (2.4%)	5 (3.8%)	2 (1.3%)	2 (1.4%)	4 (3.5%)	0.36
Maze	2 (0.4%)	2 (1.5%)	0 (0%)	0 (0%)	0 (0%)	0.09
MVR	8 (1.5%)	5 (3.8%)	0 (0%)	1 (0.7%)	2 (1.7%)	0.05
TVR	1 (0.2%)	0 (0%)	0 (0%)	1 (0.7%)	0 (0%)	0.43

AVR: aortic valve replacement; MVR: mitral valve replacement; TVR: tricuspid valve repair or replacement; PFO: Patent Foramen Ovale; LAA: left atrial appendage.

**Table 4:** Postoperative Outcomes and Complications (IPTW-Balanced).

Outcome Variable	Buckberg	Calafiore	Custodiol	STH	*p*-Value
**Sample Size**	**Weighted *n* = 131**	**Weighted *n* = 153**	**Weighted *n* = 146**	**Weighted *n* = 113**	
**Cardiac Complications**					
New-onset AF	28.5% (37)	9.3% (14)	3.8% (6)	24.7% (28)	<0.01
Arrhythmia	36.8% (48)	15.2% (23)	14.8% (22)	34.2% (39)	<0.01
AV Block II°	4.2% (6)	0% (0)	0% (0)	1.9% (2)	<0.01
AV Block III°	6.3% (8)	5.8% (9)	6.5% (9)	7.2% (8)	0.92
LBBB	1.6% (2)	1.4% (2)	3.6% (5)	0% (0)	0.18
Pacemaker implantation	4.2% (5)	11.5% (18)	6.8% (9)	6.5% (7)	0.06
Myocardial infarction	0% (0)	0% (0)	0% (0)	0% (0)	1.00
CPR	3.2% (4)	4.1% (6)	5.8% (8)	2.1% (2)	0.45
ECMO/RV failure	0% (0)	0% (0)	0.8% (1)	1.1% (1)	0.51
Impella	0% (0)	0% (0)	0.8% (1)	0% (0)	0.42
**Respiratory Complications**					
Respiratory insufficiency	16.2% (21)	7.8% (12)	8.1% (12)	14.5% (16)	0.012
**Renal Complications**					
Acute kidney injury (Stage 1–2)	12.3% (16)	10.5% (16)	11.7% (17)	13.4% (15)	0.88
New onset dialysis	0.8% (1)	2.0% (3)	3.6% (5)	0% (0)	0.14
**Neurological Complications**					
Stroke	2.3% (3)	2.1% (3)	0.9% (1)	1.2% (1)	0.65
Cerebral bleeding	0% (0)	0.7% (1)	0.7% (1)	0% (0)	0.65
Seizure	0% (0)	2.7% (4)	1.4% (2)	0.9% (1)	0.26
Delirium	10.8% (14)	6.0% (9)	5.6% (8)	11.5% (13)	0.20
Neurological deficit	4.7% (6)	3.4% (5)	1.4% (2)	5.4% (6)	0.31
**Hemorrhagic/Thrombotic**					
Major bleeding	3.1% (4)	4.0% (6)	4.2% (6)	6.4% (7)	0.64
Re-thoracotomy	3.9% (5)	4.6% (7)	3.5% (5)	1.8% (2)	0.66
Thromboembolic events	1.6% (2)	1.4% (2)	0% (0)	0.9% (1)	0.54
DVT	0% (0)	0% (0)	0% (0)	0% (0)	1.00
**Infection-related Complications**					
Sepsis	0.8% (1)	0% (0)	1.4% (2)	0.9% (1)	0.57
Wound dehiscence	2.4% (3)	1.4% (2)	1.4% (2)	2.7% (3)	0.75
Late endocarditis	2.3% (3)	0% (0)	0.7% (1)	0% (0)	0.09
**Valve-related Complications**					
Mild PVL	5.4% (7)	0% (0)	2.1% (3)	2.7% (3)	0.05
Moderate-severe PVL	0% (0)	0% (0)	0% (0)	0% (0)	1.00
Structural valve deterioration	0% (0)	0% (0)	0% (0)	0% (0)	1.00
Aortic Valve reoperation	0% (0)	0% (0)	0% (0)	0% (0)	1.00
**Other Complications**					
Vascular injury	0% (0)	0% (0)	0% (0)	0.9% (1)	0.28
**Resource Utilisation**					
ICU stay (days)	2.1 (1.0–3.8)	1.8 (1.0–3.2)	2.0 (1.0–3.5)	2.0 (1.0–3.6)	0.85
Hospital stay (days)	15.2 (10–20)	14.8 (10–19)	14.5 (10–19)	14.9 (10–19)	0.92
**Death**					
In-hospital mortality	2.8% (4)	0.9% (1)	2.3% (3)	1.1% (1)	0.42
30-day mortality	2.9% (4)	1.5% (2)	2.4% (3)	1.2% (1)	0.51
Late mortality (>30 days)	3.1% (4)	1.4% (2)	2.1% (3)	0.9% (1)	0.59

AF: atrial fibrillation; ECMO: extracorporeal membrane oxygenation; CPR: cardiopulmonary resuscitation; LBBB: left bundle branch block; DVT: deep-vein thrombosis; AV: atrioventricular block; PVL: paravalvular leak; ICU: Intensive Care Unit; RV: Right Ventricle. All percentages are weighted proportions after IPTW. Analysis performed on weighted pseudo-population after comprehensive IPTW.

**Table 5:** Postoperative echocardiographic assessments and ischemic-related labour before IPTW balancing.

Variables	Total (*N* = 543)	Buckberg (*N* = 131)	Calafiore (*N* = 153)	Custodiol (*N* = 146)	STH (*N* = 113)	*p*-Value
**Echocardiographic Data**						
Pre-OP LVEF [median (IQR)]	60 (53–66)	60 (51–65)	64 (56–70)	60 (50–65)	60 (54–65)	0.06
Post-OP LVEF [median (IQR)]	55 (50–60)	60 (48–65)	59 (50–60)	55 (49–60)	55 (50–60)	0.28
Post-OP Moderate AR	1 (0.2%)	1 (0.7%)	0 (0%)	0 (0%)	0 (0%)	0.45
Post-OP Severe AR	0 (0%)	0 (0%)	0 (0%)	0 (0%)	0 (0%)	1
Post-OP Moderate AS	8 (1.5%)	3 (2.2%)	1 (0.6%)	0 (0%)	4 (3.5%)	0.17
Post-OP Severe AS	0 (0%)	0 (0%)	0 (0%)	0 (0%)	0 (0%)	1
**Labour**						
Pre-OP CK(U/L) [median (IQR)]	111 (64–186)	111 (68–158)	115 (42–235)	115 (48–240)	96 (68–146)	0.13
Post-OP CK maximal [median (IQR)]	569 (296–800)	638 (374–1080)	325 (265–670)	430 (285–660)	684 (430–1054)	<0.01
Pre-OP CKMB [median (IQR)]	16 (13–20)	16 (13–22)	16 (12–20)	17 (12–18.5)	16.5 (14–21)	0.28
Post-OP CKMB maximal [median (IQR)]	38 (25–53)	40 (28–52.5)	28 (23–52.8)	45 (24–53)	38.5 (28.2–53)	<0.01

Pre-OP: preoperative; Post-OP: postoperative; LVEF: left ventricular ejection fraction; AR: aortic valve regurgitation; AS: aortic valve stenosis; CK: total Creatine Kinase; CK-MB: Creatine Kinase-MB.

**Table 6:** Echocardiographic Outcomes After IPTW Balancing.

Outcome Variable	Buckberg	Calafiore	Custodiol	STH	*p*-Value
**Sample Size**	**Weighted *n* = 131**	**Weighted *n* = 153**	**Weighted *n* = 146**	**Weighted *n* = 113**	
Pre-OP LVEF (%)	58.2 ± 10.5	60.8 ± 10.1	58.5 ± 11.2	59.4 ± 9.8	0.18
Post-OP LVEF (%)	54.1 ± 10.8	55.3 ± 10.2	53.8 ± 11.5	54.2 ± 9.5	0.65
Δ LVEF	−4.1 ± 3.2	−5.5 ± 2.8	−4.7 ± 3.5	−5.2 ± 3.0	0.42

**Table 7:** Myocardial Injury Biomarkers After IPTW Balancing.

Outcome Variable	Buckberg	Calafiore	Custodiol	STH	*p*-Value
**Sample Size**	**Weighted *n* = 131**	**Weighted *n* = 153**	**Weighted *n* = 146**	**Weighted *n* = 113**	
Peak CK (U/L)	920 (480–1460)	520 (320–720)	510 (330–690)	890 (450–1330)	<0.01
Peak CK-MB (U/L)	48.5 (32–65)	33.2 (24–42)	35.8 (26–46)	42.3 (30–55)	<0.01

**Table 8:** Procedural-time–adjusted sensitivity analysis (CPB and ACC times).

Variables	Total (*N* = 543)	Buckberg (*N* = 131)	Calafiore (*N* = 153)	Custodiol (*N* = 146)	STH (*N* = 113)	*p*-Value
Duration of surgery [median (IQR)]	163 (138.8–202)	198 (160.5–257)	142 (120.2–166)	161 (130.5–200)	167.5 (151–194.5)	<0.01
CPB time [median (IQR)]	84 (62–111)	112 (91–151)	61 (50–75)	81 (55–99.5)	89 (74.5–111)	<0.01
ACC time [median (IQR)]	51 (35–71)	68 (56.2–87.8)	35 (28–41)	44 (32–64)	55 (44–72.2)	<0.01
Repetition of cardioplegia [median (IQR)]	1.1 ± 0.4	1.2 ± 0.6	1.2 ± 0.5	1.0 ± 0.3	1.0 ± 0.2	<0.01
**After Balancing**						
CPB time [median (IQR)] (min)	-	90 (78–104)	89 (77–103)	91 (79–105)	90 (78–104)	0.85
ACC time [median (IQR)] (min)	-	57 (48–66)	56 (47–65)	58 (49–67)	57 (48–66)	0.92
Repetition of cardioplegia [median (IQR)]	-	1.2 (1.0–1.4)	1.1 (1.0–1.3)	1.0 (1.0–1.1)	1.0 (1.0–1.1)	<0.01
Duration of surgery [median (IQR)] (min)	-	160 (140–180)	158 (138–178)	162 (142–182)	159 (139–179)	0.88

CPB: Cardiopulmonary bypass; ACC: Aortic cross clamp.

## Data Availability

Data is provided within the manuscript or Supplementary Information files.

## Supplementary Materials

The following supporting information can be downloaded at: https://www.mdpi.com/article/10.3390/medicina62020373/s1. Table S1: Postoperative outcomes and adverse events before IPTW balancing; Table S2: Secondary analysis: restricted to isolated AVR only; Table S3: The Scheffé post hoc test.
