# Supplementary material for: Cardioplegia Strategies in Minimally Invasive Aortic Valve Replacement: An Inverse Probability of Treatment Weighting Analysis
Source: Medicina (Kaunas). 2026 Feb 13;62(2):373. doi: 10.3390/medicina62020373 (PMC12942088; doi:10.3390/medicina62020373)
Supplement: Supplementary file 1 [file medicina-62-00373-s001.zip › medicina-4097087-supplementary.pdf]

Supplemental Table S1. Post operative Outcomes and Adverse Events before IPTW Balancing

Variables	Total (N=543)	Buckberg (N=131)	Calafiore (N=153)	Custodiol (N=146)	STH (N=113)	p-value
ICU stay (days) [median (IQR)]	1 (1-2)	1 (1-1)	1 (1-2)	1 (1-3)	1 (1-1)	0.07
Hospital stay (days) [median (IQR)]	14 (11-17)	14 (11-17)	14 (11-18)	13 (10-16)	14 (11-16)	0.44
Respiratory insufficiency	61 (11.2%)	25 (19%)	10 (6.5%)	10 (6.8%)	16 (14.1%)	0.02
Aortic valve re-operation	0 (0%)	0 (0%)	0 (0%)	0 (0%)	0 (0%)	1
Arrhythmia	135 (24.8%)	51 (38.9%)	22 (14.3%)	21 (14.3%)	41 (36.2%)	0<01*
New paroxysmal AF	85 (15.7%)	41 (31.2%)	11 (7.1%)	3 (2.0%)	30 (26.5%)	0<01*
AV Block II°	8 (1.5%)	6 (4.5%)	0 (0%)	0 (0%)	2 (1.7%)	0<01*
AV Block III°	33 (6.1%)	8 (6.1%)	8 (5.2%)	9 (6.1%)	8 (7.0%)	0.22
LBBB	9 (1.7%)	2 (1.5%)	2 (1.3%)	5 (3.4%)	0 (0%)	0.18
Pacemaker implantation	39 (7.2%)	5 (3.8%)	18 (11.7%)	9 (6.1%)	7 (6.1%)	0.06
ECMO/right ventricular failure	2 (0.4%)	0 (0%)	0 (0%)	1 (0.6%)	1 (0.8%)	0.51
Impella	1 (0.2%)	0 (0%)	0 (0%)	1 (0.6%)	0 (0%)	0.43
Re-thoracotomy	19 (3.5%)	5 (3.8%)	7 (4.5%)	5 (3.4%)	2 (1.7%)	0.66
Major Bleeding	23 (4.2%)	4 (3.0%)	6 (3.9%)	6 (4.1%)	7 (6.1%)	0.64
Vascular Injuries	1 (0.2%)	0 (0%)	0 (0%)	0 (0%)	1 (0.8%)	0.28
New onset dialysis	9 (1.7%)	1 (0.7%)	3 (1.9%)	5 (3.4%)	0 (0%)	0.14
Stroke	8 (1.5%)	3 (2.2%)	3 (1.9%)	1 (0.6%)	1 (0.8%)	0.62
Cerebral bleeding	2 (0.4%)	0 (0%)	1 (0.6%)	1 (0.6%)	0 (0%)	0.65
Seizure	7 (1.3%)	0 (0%)	4 (2.6%)	2 (1.3%)	1 (0.8%)	0.26
Delirium	43 (7.9%)	14 (10.6%)	9 (5.8%)	8 (5.4%)	12 (10.6%)	0.20
Neurologic deficits	19 (3.5%)	6 (4.5%)	5 (3.2%)	2 (1.3%)	6 (5.3%)	0.31
Thromboembolic events	5 (0.9%)	2 (1.7%)	2 (1.3%)	0 (0%)	1 (0.8%)	0.54

DVT	0 (0%)	0 (0%)	0 (0%)	0 (0%)	0 (0%)	0.99
Wound dehiscence	10 (1.8%)	3 (2.2%)	2 (1.3%)	2 (1.3%)	3 (2.6%)	0.75
Sepsis	4 (0.7%)	1 (0.7%)	0 (0%)	2 (1.3%)	1 (0.8%)	0.57
Myocardial infarction	0 (0%)	0 (0%)	0 (0%)	0 (0%)	0 (0%)	0.99
Mild PVL	13 (2.4%)	7 (5.3%)	0 (0%)	3 (2.0%)	3 (2.6%)	0.05
Moderate-Severe PVL	0 (0%)	0 (0%)	0 (0%)	0 (0%)	0 (0%)	0.99
SVD	0 (0%)	0 (0%)	0 (0%)	0 (0%)	0 (0%)	0.99
Explantation of failed valve	0 (0%)	0 (0%)	0 (0%)	0 (0%)	0 (0%)	0.99
Late Endocarditis	4 (0.7%)	3 (2.3%)	0 (0%)	1 (0.7%)	0 (0%)	0.09
CPR	20 (3.7%)	4 (3.0%)	6 (3.9%)	8 (5.4%)	2 (1.7%)	0.45
In-hospital mortality	9 (1.7%)	4 (3.0%)	1 (0.6%)	3 (2.0%)	1 (0.8%)	0.38
30-day mortality	10 (1.8%)	4 (3.0%)	2 (1.3%)	3 (2.0%)	1 (0.8%)	0.59
Late mortality	10 (1.8%)	4 (3.0%)	2 (1.3%)	3 (2.0%)	1 (0.8%)	0.59

CPB: Cardiopulmonary bypass; AF: Atrial Fibrillation; FFP: Fresh Frozen Plasma; ECMO: Extracorporeal membrane oxygenation; CPR: Cardiopulmonary resuscitation; SVD: Structural Valve Deterioration; LBBB: Left bundle branch block; DVT: Deep vein thrombosis; AV; atrioventricular block; PVL: Paravalvular leak
* p-value significant

Supplemental Table S2. Secondary Analysis: Restricted to Isolated AVR Only

A. Sample Characteristics After Restriction

Variables	Buckberg (N=131)	Calafiore (N=153)	Custodiol (N=146)	STH (N=113)	p-value
**Isolated AVR**	118 (90.1%)	151 (98.7%)	142 (97.3%)	107 (94.7%)	**0.001***
**AVR with Concomitant Procedures**	13 (9.9%)	2 (1.3%)	4 (2.7%)	6 (5.3%)	**0.001***
Concomitant procedures include:					
- Mitral valve repair/replacement	5 (3.8%)	0 (0%)	1 (0.7%)	2 (1.8%)	
- Tricuspid valve repair	0 (0%)	0 (0%)	1 (0.7%)	0 (0%)	
- Left atrial appendage closure	5 (3.8%)	2 (1.3%)	2 (1.4%)	4 (3.5%)	
- Maze procedure	2 (1.5%)	0 (0%)	0 (0%)	0 (0%)	
- Patent foramen ovale closure	1 (0.7%)	0 (0%)	0 (0%)	0 (0%)	

B. Operative Efficiency (Isolated Cases Only):

Parameter	Buckberg (N=118)	Calafiore (N=151)	Custodiol (N=142)	STH (N=107)	p-value
**ACC time (min)**	72 (50-89)	37 (28-46)	52 (38-65)	58 (45-72)	**<0.001**
**CPB time (min)**	120 (92-148)	64 (52-76)	88 (68-108)	92 (74-110)	**<0.001**
**Total OR time (min)**	195 (158-232)	132 (120-144)	148 (120-176)	152 (132-172)	**<0.001**

C. Clinical Outcomes (Isolated Cases Only):

Outcome	Buckberg (N=118)	Calafiore (N=151)	Custodiol (N=142)	STH (N=107)	p-value
**NOAF (%)**	29.7%	7.3%	2.1%	25.2%	**<0.001**
**Peak CK (U/L)**	985 (420-1560)	460 (280-640)	475 (310-640)	920 (380-1460)	**<0.001**
**Respiratory insufficiency**	17.8%	6.0%	6.3%	13.1%	**0.015**
**30-day mortality**	2.5%	1.3%	2.1%	0.9%	0.68

Supplemental Table S3. The Scheffé post hoc test

Variable				Mean Difference	Std. Error	Sig.	CI 95% Lower Bound	CI 95% Upper Bound
Postoperative CK maximal	Scheffe	Buckberg	Calafiore	597.3	146.6	0.001	185.9	1008.6
			Custodiol	592.2	184.0	0.017	75.7	1108.7
			STH	104.5	158.1	0.932	-339.0	548.2
		Calafiore	Buckberg	-597.3	146.6	0.001	-1008.6	-185.9
			Custodiol	-5.0	179.5	1.000	-508.7	498.6
			STH	-492.7	152.7	0.016	-921.3	-64.0
		Custodiol	Buckberg	-592.2	184.0	0.017	-1108.7	-75.7
			Calafiore	5.0	179.5	1.000	-498.6	508.7
			STH	-487.6	189.0	0.085	-1018.0	42.7
		STH	Buckberg	-104.5	158.1	0.932	-548.2	339.0
			Calafiore	492.7	152.7	0.016	64.0	921.3
			Custodiol	487.6	189.0	0.085	-42.7	1018.0

Post-hoc pairwise comparisons of peak post-operative CK levels revealed significant differences between several cardioplegia groups. The Buckberg group demonstrated significantly higher peak CK levels compared with the Calafiore group (mean difference = 597.3 U/L, 95% CI 185.9–1008.6, $p = 0.001$) and the Custodiol group (mean difference = 592.2 U/L, 95% CI 75.7–1108.7, $p = 0.017$). Similarly, St. Thomas cardioplegia showed significantly higher peak CK levels compared with Calafiore (mean difference = 492.7 U/L, 95% CI 64.0 to 921.3, $p = 0.016$). No other pairwise comparisons reached statistical significance ($p > 0.05$).

Postoperative CK MB maximal	Scheffe	Buckberg	Calafiore	20.8	5.9	0.008	3.9	37.6
			Custodiol	15.2	7.5	0.254	-5.9	36.3
			STH	11.7	6.4	0.345	-6.3	29.9
		Calafiore	Buckberg	-20.8	5.9	0.008	-37.6	-3.9
			Custodiol	-5.6	7.3	0.900	-26.1	14.9

			STH	-9.0	6.2	0.555	-26.5	8.4
		Custodiol	Buckberg	-15.2	7.5	0.254	-36.3	5.9
			Calafiore	5.6	7.3	0.900	-14.9	26.1
			STH	-3.4	7.7	0.978	-25.0	18.2
		STH	Buckberg	-11.7	6.4	0.345	-29.9	6.3
			Calafiore	9.0	6.2	0.555	-8.4	26.5
			Custodiol	3.4	7.7	0.978	-18.2	25.0

Post-hoc pairwise comparisons of peak post-operative CK-MB levels demonstrated that the Buckberg group had significantly higher CK-MB values than the Calafiore group (mean difference = 20.8 U/L, 95% CI 3.9–37.6, p = 0.008). No other intergroup comparisons reached statistical significance (all p > 0.05).

Duration of surgery (Hours:Minutes)	Scheffe	Buckberg	Calafiore	1:07	0:06	0.001	0:48	1:25
			Custodiol	0:35	0:06	0.001	0:17	0:54
			STH	0:34	0:07	0.001	0:14	0:54
		Calafiore	Buckberg	-1:07	0:06	0.001	-1:25	-0:48
			Custodiol	-0:31	0:06	0.001	-0:49	-0:13
			STH	-0:33	0:06	0.001	-0:52	-0:13
		Custodiol	Buckberg	-0:35	0:06	0.001	-0:54	-0:17
			Calafiore	0:31	0:06	0.001	0:13	0:49
			STH	-0:01	0:06	0.997	-0:21	0:17
		STH	Buckberg	-0:34	0:07	0.001	-0:54	-0:14
			Calafiore	0:33	0:06	0.001	0:13	0:52
			Custodiol	0:01	0:06	0.997	-0:17	0:21

Post-hoc pairwise comparisons of operative duration demonstrated that surgeries performed with Buckberg cardioplegia were significantly longer than those in all other groups. The Buckberg group had a mean operative time 1 hour 07 minutes longer than Calafiore (95% CI 0:48–1:25, p = 0.001), 35 minutes longer than Custodiol (95% CI 0:17–0:54, p = 0.001), and 34 minutes longer than St. Thomas (95% CI 0:14–0:54, p = 0.001). Conversely, Calafiore procedures were significantly shorter than both Buckberg and Custodiol (mean difference = –0:31 hours, 95% CI –0:49 to –0:13, p = 0.001) and St. Thomas (mean difference = –0:33 hours, 95% CI –0:52 to –0:13, p = 0.001). No significant difference was observed between Custodiol and St. Thomas operative times (p = 0.997).

Time on CPB (Minutes)	Scheffe	Buckberg	Calafiore	58.2	4.7	0.001	44.8	71.5
			Custodiol	33.0	6.1	0.001	15.8	50.3
			STH	28.9	5.1	0.001	14.5	43.4
		Calafiore	Buckberg	-58.2	4.7	0.001	-71.5	-44.8
			Custodiol	-25.1	6.0	0.001	-41.9	-8.2
			STH	-29.2	4.9	0.001	-43.1	-15.3
		Custodiol	Buckberg	-33.0	6.1	0.001	-50.3	-15.8
			Calafiore	25.1	6.0	0.001	8.2	41.9
			STH	-4.1	6.3	0.935	-21.8	13.6
		STH	Buckberg	-28.9	5.1	0.001	-43.4	-14.5
			Calafiore	29.2	4.9	0.001	15.3	43.1
			Custodiol	4.1	6.3	0.935	-13.6	21.8

Post-hoc pairwise comparisons of cardiopulmonary bypass (CPB) duration (Scheffé test) demonstrated that the Buckberg group had significantly longer CPB times than all other groups. CPB time was 58.2 minutes longer compared with Calafiore (95% CI 44.8–71.5, p = 0.001), 33.0 minutes longer than Custodiol (95% CI 15.8–50.3, p = 0.001), and 28.9 minutes longer than St. Thomas (95% CI 14.5–43.4, p = 0.001). Conversely, Calafiore had significantly shorter CPB times than both Custodiol (mean difference = –25.1 minutes, 95% CI –41.9 to –8.2, p = 0.001) and St. Thomas (mean difference = –29.2 minutes, 95% CI –43.1 to –15.3, p = 0.001). No significant difference was observed between Custodiol and St. Thomas groups (p = 0.935).

Aortic Cross Clamping Time	Scheffe	Buckberg	Calafiore	36.3	3.0	0.001	27.6	44.9
			Custodiol	19.6	3.9	0.001	8.4	30.8
			STH	14.0	3.3	0.001	4.7	23.3
		Calafiore	Buckberg	-36.3	3.0	0.001	-44.9	-27.6
			Custodiol	-16.6	3.8	0.001	-27.6	-5.7
			STH	-22.2	3.1	0.001	-31.2	-13.3
		Custodiol	Buckberg	-19.6	3.9	0.001	-30.8	-8.4
			Calafiore	16.6	3.8	0.001	5.7	27.6
			STH	-5.5	4.0	.602	-17.0	5.8
		STH	Buckberg	-14.0	3.3	0.001	-23.3	-4.7
			Calafiore	22.2	3.1	0.001	13.3	31.2
			Custodiol	5.5	4.0	.602	-5.8	17.0

Post-hoc pairwise comparisons of ACC time with Scheffé test showed that the Buckberg group had significantly longer ACC times than all other groups. ACC time was 36.3 minutes longer than Calafiore (95% CI 27.6–44.9, p = 0.001), 19.6 minutes longer than Custodiol (95% CI 8.4–30.8, p = 0.001), and 14.0 minutes longer than St. Thomas (95% CI 4.7–23.3, p = 0.001). Calafiore demonstrated significantly shorter ACC times than both Custodiol (mean difference = –16.6 minutes, 95% CI –27.6 to –5.7, p = 0.001) and St. Thomas (mean difference = –22.2 minutes, 95% CI –31.2 to –13.3, p = 0.001). No significant difference was found between Custodiol and St. Thomas groups (p = 0.602).

Repetition of cardioplegia	Scheffe	Buckberg	Calafiore	-0.03	.053	.951	-.018	0.12
			Custodiol	0.1	.054	.005	0.04	0.35
			STH	0.2	.058	.004	0.05	0.37
		Calafiore	Buckberg	0.03	0.05	0.951	-0.12	0.18
			Custodiol	0.2	0.05	0.001	0.08	0.37
			STH	0.2	0.05	0.001	0.09	0.40
		Custodiol	Buckberg	-0.1	0.05	0.005	-0.35	-0.04
			Calafiore	-0.2	0.05	0.001	-0.37	-0.08
			STH	0.01	0.05	0.995	-0.14	0.17
		STH	Buckberg	-0.2	0.05	0.004	-0.37	-0.05
			Calafiore	-0.2	0.05	0.000	-0.40	-0.09
			Custodiol	-0.01	0.05	0.995	-0.17	0.14

Post-hoc pairwise comparisons for the number of cardioplegia repetitions (Scheffé test) revealed that Buckberg and Calafiore groups required significantly more repeated doses of cardioplegia than Custodiol and St. Thomas groups. Buckberg required on average 0.1 more doses than Custodiol (95% CI 0.04–0.35, p = 0.005) and 0.2 more doses than St. Thomas (95% CI 0.05–0.37, p = 0.004). Similarly, Calafiore required 0.2 more doses than Custodiol (95% CI 0.08–0.37, p = 0.001) and 0.2 more than St. Thomas (95% CI 0.09–0.40, p < 0.001). No significant differences were observed between Buckberg and Calafiore (p = 0.951) or between Custodiol and St. Thomas (p = 0.995).
